# Impact of early neonatal nutrition on neurodevelopment and the limbic system in very low birth weight infants

**DOI:** 10.1017/S0007114525105874

**Published:** 2026-02-14

**Authors:** Jose Uberos, Marta Carrasco-Solis, Carolina Laynez-Rubio, Ana Nieto-Ruiz, Aida Ruiz-López, Francisco Contreras-Chova, Elizabeth Fernández-Marin, Ana Campos-Martínez, Collaborative NeuroPrem group

**Affiliations:** 1 Department of Paediatrics, School of Medicine, Granada University, Granada, Spain; 2 Neonatal Intensive Care Unit, San Cecilio Clinical Hospital, Medicine Faculty, Granada, Spain; 3 Neuropaediatrics Unit, Paediatrics Service, San Cecilio Clinical Hospital, Granada, Spain; 4 Department of Behavioural Science Methodology, Faculty of Psychology, University of Granada, Granada, Spain; 5 EURISTIKOS Excellence Centre for Paediatric Research, Biomedical Research Centre, Department of Paediatrics, Faculty of Medicine, University of Granada, Granada, Spain; 6 Instituto de Investigación Biosanitaria (ibs.GRANADA), Health Sciences Technological Park, Granada, Spain; 7 Center of Psychology and Neuropsychology (a)LPEH, Granada, Spain

**Keywords:** Limbic system, Nutrition, Thalamus, Nucleus accumbens, Very low birth weight infants

## Abstract

The limbic system is a brain structure involved in emotional regulation. Since nutritional interventions in very low birth weight (VLBW) infants may be associated with measurable differences in brain structure and function, we designed this prospective study to evaluate the impact of early nutritional support in VLBW infants on the volume of the regions that comprise the limbic system, as well as on the emotional and neuropsychological development of these infants. This is a prospective observational study of a historical cohort of children with a history of prematurity. Seventy-four preterm infants, with a mean age of 11·1 (sd 2·9) years, underwent neuropsychological assessment using the Wechsler Intelligence Scale for Children and functional MRI (fMRI). We recorded the nutritional intake during the first week of the neonatal period, as well as data related to neonatal morbidity. The association between the results of the brain structural analysis, psychometrics variables and nutritional intake was determined using simple and multivariate linear regression adjusted for child age and BMI in the structural analysis of fMRI. Lipids intake was also associated with the volume of the left thalamus (*b* = 50·7; *P* = 0·014), the right thalamus (*b* = 47·4; *P* = 0·018) and the left nucleus accumbens (*b* = 5·02; *P* = 0·031). We conclude that lipids intake in the first week of life in VLBW newborns is associated with the volume of various structures of the limbic system, namely the thalamus and the nucleus accumbens.

When childbirth is very premature (< 32 weeks gestational age), the brain is still at a critical stage of development, including the active growth of deep nuclear grey matter^(1)^. In phylogenetic terms, the limbic system is one of the oldest parts of the brain, and its constituent structures are involved in the integration of emotional and behavioural processes. The limbic system comprises various structures that contribute to the integration of emotional processes, memory formation, motivation, initiative and learning^(2)^. Subcortical limbic structures include the hippocampus, the cingulate cortex, the amygdala, the parahippocampal cortex, parts of the thalamus and the ventral striatum (i.e. the nucleus accumbens)^(3)^. The cingulate cortex is the meeting point for processes such as memory, cognition, affection and others involved in the complex phenomena of pain experience, memory, spatial functioning, reward, cognition, emotion and visceromotor and endocrine control^(4)^.

Nutritional interventions in very low birth weight (VLBW) infants are associated with measurable differences in early brain structure and function. In earlier research, a higher intake of breast milk has been associated with a larger volume of cortical grey matter, including the cingulate cortex and of deep nuclear grey matter^(5)^. These findings are consistent with a growing body of evidence indicating that very early macronutrient intake has long-term effects on brain development in the premature infant and on functional outcomes at school age^(6)^. Moreover, protein and energy intake during the first week after birth predicts better cognitive outcomes at 18 months, according to a study of extremely low birth weight infants^(7)^. In other studies of very premature infants who underwent serial MRI from birth, total energy, carbohydrate, lipid and protein intake in the first weeks of life predicted larger brain and caudate nucleus volumes at term-equivalent age, as well as better cognitive outcomes at 18 months^(8)^.

Other studies, however, have reported mixed results. For some, DHA supplementation does not produce significant cognitive or neuroanatomical changes, while others report improvements in emotional and language development at three years of age^(9)^. Overall, studies of early life outcomes indicate that nutritional interventions may favourably influence brain regions that contribute to limbic system function^(10)^. To our knowledge, however, no studies have yet been conducted on the relationship between very early macronutrient intake in highly preterm infants, brain connectivity and neurodevelopmental outcomes at school age, which is when different aspects of brain function can be more reliably assessed.

According to the sociobiological vulnerability model of development, first proposed by Healy *et al.* ^(11)^, adolescents who were born prematurely are more likely to experience problems of socialisation, due to structural and functional changes in specific brain networks that can generate deficits in cognition and socio-emotional functioning. As a result, children born prematurely are more vulnerable and at greater risk of negative social experiences, such as bullying and exclusion, which have been associated with increased stress-induced dopamine release in the striatum and with dopamine sensitisation in mesolimbic areas^(12)^.

Due to the increased risk of mental and emotional processing disorders, including autism spectrum disorders, in children born prematurely, it seems important to include these children in early assessments of psychological development and to study the perinatal factors that may influence neurological outcomes. In our opinion, nutritional approaches represent a necessary and modifiable approach to these neurodevelopmental deficits.

Latal-Hajnal *et al.* ^(13)^ have demonstrated that postnatal growth is an independent factor for adverse neurodevelopmental outcomes in preterm infants. Specifically, the pattern of postnatal growth is associated to varying degrees with neurodevelopment during the first years of life^(14)^. Adolescents born prematurely often experience emotional and behavioural challenges, including higher levels of anxiety, depression and introversion compared to their full-term peers. They have been reported to have difficulties managing emotions and forming friendships, exhibiting more intense and volatile moods^(12)^. These problems, which could be due to biological factors related to their premature birth, as well as environmental factors such as parental stress and support, may also have a structural basis in the development of certain brain regions during the perinatal period^(14)^. We propose the working hypothesis that some of these emotional changes may also be related to early nutritional status during the perinatal period and structural modifications of the limbic system during critical periods of development.

In view of these considerations, the main objective of the present study is to evaluate the impact of early nutritional support on neuropsychological development in VLBW premature newborns, as assessed by the Weschsler test at school age and on brain volumes in the regions comprising the limbic system.

## Methods

### Study design and subjects

A prospective study was conducted of a cohort of VLBW premature infants admitted to a neonatal intensive care unit during the period January 2008 to December 2017. For these newborns, obstetric data are recorded, including type of delivery, presence of chorioamnionitis, duration of ruptured membranes, maternal antibiotic treatment and any maternal hypertension or diabetes. The types and amounts of enteral and parenteral nutrition administered, as well as daily anthropometric measurements, are also recorded. Any comorbidities that develop in the newborns are also documented. From March 2023 to March 2025, all children in the study cohort will be offered the opportunity to participate in the study, which involves a brain fMRI scan and a neuropsychological assessment, details of which are provided below. Of the 402 newborns initially considered, fifty-five died within the first month of life. This study was conducted according to the guidelines laid down in the Declaration of Helsinki, and all procedures involving human subjects/patients were approved by the Granada (Spain) Bioethics Committee (Code: 6hWMS879PFIRMAbeKXdHsslK2Ive5Z) on 1 February 2023). Written informed consent was obtained from all subjects/patients. Parents were asked to provide written general informed consent for their child’s participation in the study, and specific consent for the fMRI procedure was obtained from both the parents and the child for those over 12 years of age.

All current regulations regarding data confidentiality were respected.

#### Inclusion and exclusion criteria

**Figure 1.**
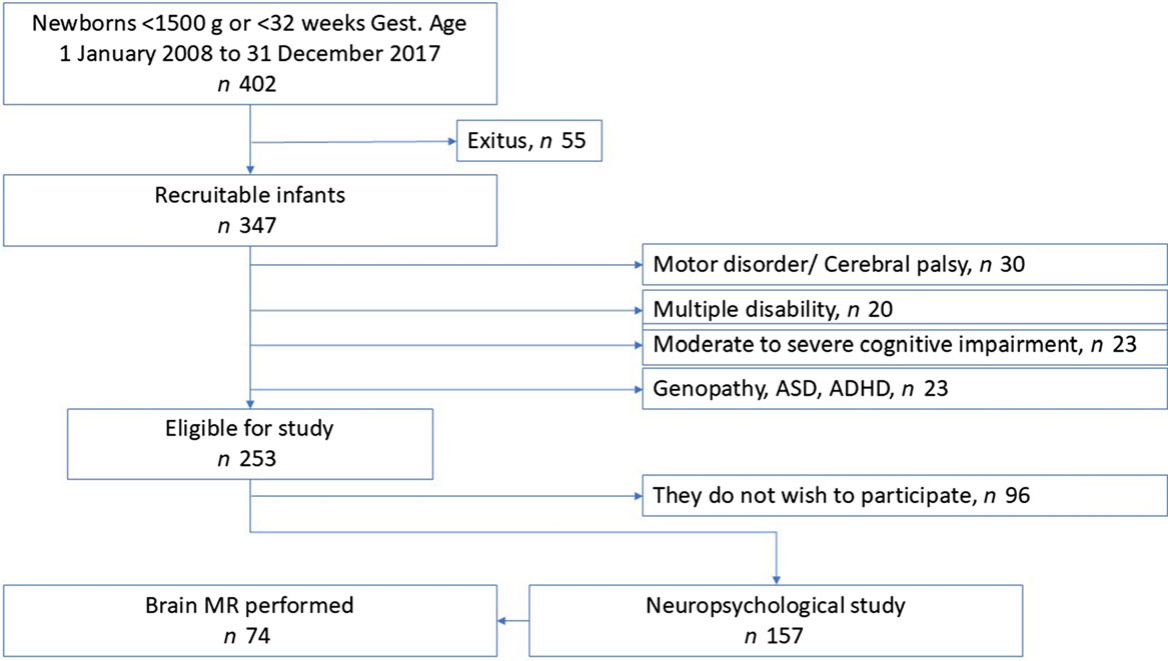
Flow diagram for the study cohort.

The following children with moderate or severe disability were excluded: thirty with cerebral palsy or severe motor disabilities, twenty with multiple disabilities, twenty-three with moderate to severe cognitive delays and twenty-three with genopathies, severe autism spectrum disorder or attention deficit hyperactivity disorder, diagnosed and under treatment. The reasons for excluding these patients are related to the inability to perform and complete neuropsychological assessments, as well as the difficulty, in many cases, of conducting functional magnetic resonance imaging (fMRI) without sedating the patient. All of these patients, with varying degrees of disability, receive follow-up care in our paediatric neurology clinic. Finally, thus, 253 children were considered eligible for the study. However, on being asked to provide informed consent, ninety-six chose not to participate. A neuropsychological evaluation based on psychometric tests was performed of the remaining 157 children, and functional magnetic resonance imaging, without sedation, was performed on seventy-four of these (Figure 1).

### Data collection and assessment

#### Anthropometry

The body weight, length and head circumference of each newborn were retrospectively recorded, and z-scores were calculated using Fenton growth charts^(15)^. The diagnosis of fetal growth restriction was made in accordance with the 2016 Delphi international consensus criteria^(16)^ and includes an estimated fetal weight or abdominal circumference < 3rd percentile or < 10th percentile combined with abnormal Doppler findings. In our sample, all fetal growth restriction cases were classified as small for gestational age at birth. In accordance with the classification proposed by Alexander *et al.* ^(17)^, small for gestational age is defined as birth weight < 10th percentile for gestational age.

#### Nutritional management

The overall nutritional strategy and the liquid intake supplied to the newborn were in accordance with the standard protocol of our neonatal unit and with the recommendations of the Nutrition and Metabolism Group of the Spanish Society of Neonatology^(18,19)^. During the study period, certain changes were made to the usual nutritional practice. On the one hand, from April 2011, first-day parenteral nutrition (early parenteral nutrition) was supplied as an amino acid solution (2 g/kg) and glucose at 5 mg/kg/min. Prior to this date, amino acids were not provided during the first hours of life. This change, which aligned our treatment protocols with international recommendations, increased the energy intake of newborns in the first week of life and, secondarily, allowed us to more effectively assess its impact. The second modification concerned the lipid emulsion used. In July 2016, SMOFlipid (Fresenius Kabi) was introduced, replacing Intralipid (Fresenius Kabi) as the standard emulsion for parenteral nutrition. The impact of this change on energy intake is taken into account in our analysis of the results.

The usual procedure during the newborn’s first days of life is to supplement enteral nutrition with parenteral nutrition when full enteral nutrition cannot be established. Daily liquid, protein and lipid requirements are calculated daily. In our hospital, breast milk composition is determined according to the Standardised Neonatal Growth and Nutrition Report checklist, and formula composition is assessed according to commercial reports^(20)^. In all cases, the clinical aim is to meet the minimum nutritional requirements to ensure adequate growth during the first week of life, according to standard recommendations^(21)^. For the present study, liquid, energy, protein, carbohydrate and lipid intakes were recorded during the first week of life.

Since January 2008, the neonatal intensive care unit has prospectively recorded the daily enteral nutrition and parenteral nutrition intake of all VLBW patients, using an Excel database, specifying the intake per kg of body weight of energy, protein, carbohydrates and lipids. The timing, type and volume of enteral nutrition are also recorded.

#### Neonatal morbidity

The following retrospective records of neonatal morbidity were obtained.

Bronchopulmonary dysplasia, defined in accordance with the classification proposed by Jobe and Bancalari and the NIHCD^(22,23)^, as a need for supplemental oxygen > 21 % at 28 d of life and/or a need for supplemental oxygen > 21 % or for positive airway pressure at 36 weeks’ corrected gestational age.

Persistent ductus arteriosus, diagnosed by Doppler ultrasound and treated when clinical repercussions are observed or when the diameter is greater than 2 mm.

Clinical sepsis, diagnosed when an NOSEP-1 score > 8 is recorded. On this scale, the presence of C-reactive protein ( > 0·014 g/l) is assigned five points; that of neutrophils > 50 %, three points; that of thrombocytopaenia < 150 × 10^9^/l, five points; and that of fever > 38·2℃, five points^(24)^. The clinical risk index for babies (CRIB II score) for each newborn was performed using the following variables: sex, gestational age (in weeks), birth weight (in grams) and excess base. The total CRIB II score (range 0–27) was calculated^(25)^.

Retinopathy of prematurity, diagnosed and staged following a retinal examination before discharge from the neonatal unit^(26,27)^.

Intraventricular haemorrhage, diagnosed according to Papile’s classification^(28)^. All neonates in this study received a transfontanellar ultrasound examination on the third day of life and every week thereafter during admission.

Necrotising enterocolitis, diagnosed and classified according to Bell’s criteria^(29)^. Cases classified as spontaneous intestinal perforations were excluded from this diagnosis.

#### Cognitive performance

Assessments of the 157 children began in March 2023 and continued until December 2024. During the first evaluation session, children underwent an evaluation using the Wechsler Intelligence Scale for Children – Fifth Edition (WISC-V; Pearson, 2014). This test provides five composite scores that summarise cognitive main abilities in different areas covering: verbal comprehension, visual spatial, fluid reasoning, working memory and processing speed. All these indexes can be combined to provide a full scale intelligence quotient. During the assessment, the quantitative reasoning, which is a secondary index of the scale that merges fluid reasoning and arithmetics scores was included. The composite scores of each subtest were used as the main measure of cognitive assessment.

#### MRI data acquisition and neuroimage processing

Prior to the neuroimaging session, the children were familiarised with the sounds of the scanner and with the MRI environment. Brain data and images were acquired with a 3 T MRI scanner equipped with a thirty-two channel receiving phased array head coil (Magnetom Trio Siemens Medical System, ERLANGEN, Germany), at the Mind, Brain and Behaviour Research Centre (CIMCYC) of the University of Granada, Spain. A high-resolution T1-weighted 3D magnetisation-prepared rapid gradient-echo sequence was acquired for each participant, with the following parameters: repetition time 2·3 ms, echo time 3·1 ms, flip angle 9°, field of view 256 × 256 mm, matrix size 320 × 320, number of slices 208. With these parameters, an isotropic resolution of 0·8 × 0·8 × 0·8 mm was obtained. Total acquisition time for the T1 sequence was 6 min 35 sec. Head movements were minimised using a foam system fitted around the participant’s head. Furthermore, a cartoon film was projected to reassure the child during the MRI scanning.

**Figure 2.**
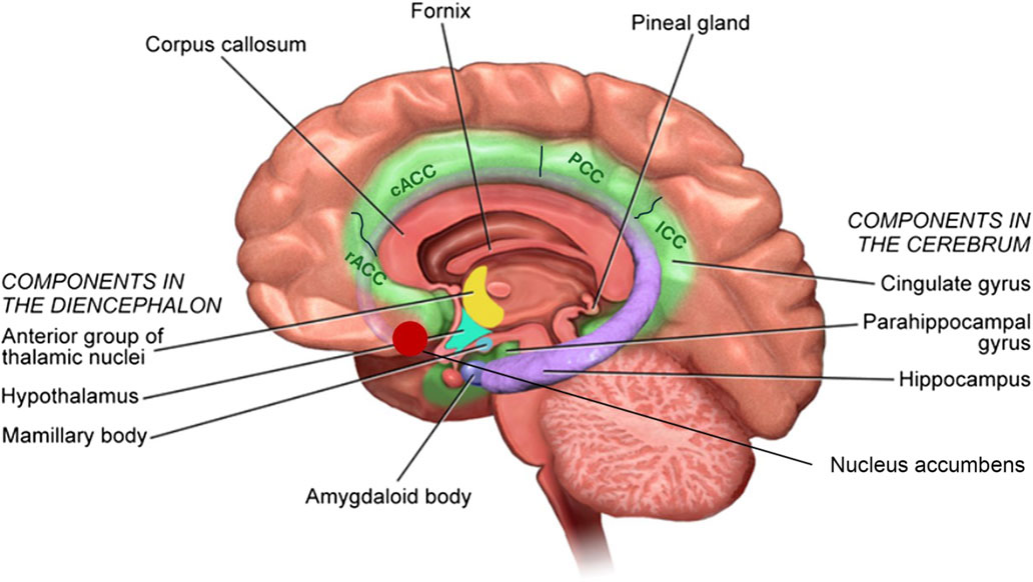
Anatomical representation of the areas and regions of the limbic system. In this study, we distinguish the rostral anterior cingulate cortex (rACC), the caudal anterior cingulate cortex (cACC), the posterior cingulate cortex (PCC), the isthmus cingulate cortex (ICC), the parahippocampal cortex (PHC), the thalamus (Th), the amygdala (Amg), the hippocampus (Hipp) and the nucleus accumbens (NAc).

All images were visually inspected for major artifacts and realigned to the AC-PC line. Image processing was performed using the automated processing ‘*recon-all*’ pipeline in FreeSurfer software (version 6.0, http://surfer.nmr.mgh.harvard.edu/) on the Alhambra Cluster of the University of Granada (Spain). Preprocessing steps involved intensity normalisation, registration to the Talairach space, skull stripping, segmentation of white matter, tessellation of the white matter boundary and automatic correction of topological defects. The cerebral cortex was then parcelled into regions of interest based on gyral and sulcal structures from the Destrieux atlas^(30–32)^. FreeSurfer outputs were also visually inspected to check for correct segmentation and parcellation. In addition, a segmentation was performed of the rostral anterior cingulate cortex, the caudal anterior cingulate cortex, the posterior cingulate cortex, the isthmus cingulate cortex, the parahippocampal cortex, the thalamus, the amygdala, the hippocampus and the nucleus accumbens (Figure 2).

#### Statistical analysis

We verified that the study variables follow a normal distribution using the Kolmogorov–Smirnov test. The continuous variables were categorised by their means and sd, and the categorical variables, by the frequency distribution. Comparative analyses were performed using a *t* test for the continuous variables and a *χ* ^2^ test for the categorical variables. In addition, a Pearson correlation study of the variables was performed. The association between the results of the brain structural analysis, the psychometric variables and the nutritional intake was determined using simple and multivariate linear regression adjusted for child age and BMI in the structural analysis of fMRI. *P* values < 0·05 were considered statistically significant. All statistical analyses were performed using IBM SPSS 22.0 for Windows (IBM).

#### Reporting

The STROBE checklist for reporting observational studies was used^(30)^.

## Results

The study analysis focused on a cohort of seventy-four children born prematurely at our hospital between January 2008 and December 2017, with a mean age of 11·1 (sd 2·9) years. The children had a history of VLBW, a mean age of 11·0 years (95 % CI 8·2, 13·7) and a BMI of 18·1 kg/m^2^ (95 % CI 15·5, 20·3). After obtaining informed consent, a complete neurodevelopmental evaluation was performed, with psychometric testing and a structural brain study using functional magnetic resonance imaging.

As shown in Table 1, the children had a mean gestational age of 29·2 (sd 1·9) weeks, with a minimum of 24 weeks and a maximum of 32 weeks. The mean weight at birth was 1234 (sd 346) g, with a minimum of 535 g and a maximum of 2220 g. Fetal growth restriction and low birth weight were experienced by 16·2 % of the children, and 59·4 % were breastfed from the first days of life. Grade 1 intraventricular haemorrhage was present in 12·1 % of cases and Grade 2 necrotising enterocolitis in 25·6 %, while 16·2 % of the children had a history of late neonatal sepsis.

**Table 1.** Gestational, neonatal and childhood characteristics of the study cohort

	Mean/*n*	sd/%
	*n* 74	
	*n*	%
PIH	6	8·1
Chorioamnionitis	9	12·1
Prenatal antibiotics	32	43·2
Prenatal glucocorticoids	68	91·8
PPROM	21	28·3
	Mean	sd
Gestation (weeks)	29·2	1·9
	*n*	%
Gestation ≤ 27 weeks	17	22·9
FGR	12	16·2
Twin birth	28	37·8
Caesarean section	63	85·1
Mother’s milk feeding	44	59·4
Neonatal		
	Mean	sd
Birth weight (g)	1234	346
	*n*	%
Birth weight (z-score)	–0·31	0·88
Male sex	33	44·5
Apgar < 5 at 5 min	5	6·7
BPD	26	35·1
PDA	14	18·9
IVH (Grade 1–2)	9	12·1
IVH (Grade 3)	1	1·3
NEC (Grade ≥ 2)	19	25·6
Late onset sepsis	12	16·2
	Mean	sd
Childhood		
Age (years)	11·1	2·9
Weight (kg)	40·0	16·0
Height (cm)	145·1	18·2
Body mass index (kg/m^2^)	18·2	3·6
	*n*	%
Socio-economic status		
Mother’s education background		
Higher education	26	35·1
Secondary education	8	10·8
Father’s education background		
Higher education	16	21·6
Secondary education	12	16·2
Received early childhood care	43	58·1

Mean (sd); *n* (%). PIH, pregnancy-induced hypertension; PPROM, preterm pre-labour rupture of membranes; FGR, fetal growth restriction; BPD, bronchopulmonary dysplasia; PDA, persistent ductus arteriosus; IVH, intraventricular haemorrhage; NEC, necrotising enterocolitis.

The average energy intake during the first week of life was 63·5 kcal/kg/d (or 445 kcal/kg/w). The basal energy requirements in the first week of life are estimated at 50 kcal/kg/d^(31)^. With respect to energy intake in the first week of life, the first quartile of our cohort (*n* 22) received < 50 kcal/kg/d. Protein intake in the first quartile of our distribution remained above the basal requirement of 1 g/kg/d, although 2·5 g/kg/d is necessary to meet growth requirements^(31)^. In our cohort, the mean protein intake was 2·2 g/kg/d. The mean fat intake was 12·2 g/kg/w, which represents an average daily intake of 1·7 g/kg/d. (Table 2).

**Table 2.** Nutritional intake of the cohort of VLBW premature newborns during the first week of life

	Quartile 1	Quartile 2	Quartile 3	Quartile 4
Liquids ml/kg/w	< 760·0	760·0–846·7	846·7–904·1	> 904·1
Energy kcal/kg/w	< 387·3	387·3–465·9	465·9–521·4	> 521·4
Proteins g/kg/w	< 9·74	9·74–15·8	15·8–19·1	> 19·1
Carbohydrates g/kg/w	< 62·3	62·3–72·0	72·0–79·0	> 79·0
Lipids g/kg/w	< 9	9–12·5	12·5–15·2	> 15·2

We initially observed a significant association between energy intake in the first week of life and the volume of the left posterior cingulate cortex (*b* = 1·53; *P* = 0·006). After adjusting for the child’s chronological age and BMI, the association becomes non-significant (*b* = 1·09; *P* = 0·109) (Table 3). Protein intake in the first week of life was significantly associated initially with the volume of the isthmus of the right cingulate cortex (*b* = 22·1; *P* = 0·023). After adjusting for the child’s chronological age and BMI, the association becomes non-significant (*b* = 8·4; *P* = 0·544) (Table 4).

**Table 3.** Regression analysis of energy intake (kcal/kg/w) and volume (mm^3^) of the cortical and subcortical regions within the limbic system in the first week of life, for school-aged children with a history of prematurity. Regression adjusted for child age and body mass index in the structural analysis of MRf

Variable (mm^3^)	Not adjusted				Adjusted			
	b	se	R^2^	*P*	b	se	R^2^	*P*
rACC – L	0·662	0·654	0·014	0·314	0·62	0·81	0·029	0·445
rACC – R	–0·075	0·625	0·000	0·905	–0·14	0·78	0·005	0·850
cACC – L	0·525	0·525	0·014	0·320	0·39	0·65	0·032	0·549
cACC – R	0·446	0·734	0·005	0·546	0·35	0·92	0·006	0·698
PCC – L	1·536	0·546	0·099	**0·006**	1·09	0·67	0·116	0·109
PCC – R	1·184	0·607	0·050	0·055	0·54	0·74	0·095	0·467
ICC – L	0·297	0·599	0·003	0·622	–0·61	0·72	0·067	0·402
ICC – R	0·812	0·487	0·037	0·100	0·11	0·59	0·089	0·846
PHC – L	0·007	0·375	0·000	0·986	1·09	0·67	0·116	0·109
PHC – R	0·182	0·366	0·003	0·621	0·54	0·74	0·095	0·467
Th – L	1·326	0·925	0·028	0·156	2·04	1·14	0·052	0·078
Th – R	1·269	0·897	0·027	0·162	1·79	1·11	0·050	0·110
Amg – L	0·161	0·244	0·006	0·512	0·19	0·30	0·007	0·517
Amg – R	0·294	0·263	0·017	0·267	0·29	0·32	0·018	0·371
Hipp – L	0·332	0·448	0·008	0·461	0·73	0·55	0·030	0·191
Hipp – R	0·713	0·485	0·029	0·146	0·93	0·60	0·038	0·127
NAc – L	0·197	0·109	0·043	0·075	0·16	0·13	0·047	0·232
NAc – R	0·129	0·105	0·021	0·223	0·13	0·13	0·029	0·310

L, left hemisphere; R, right hemisphere; rACC, rostral anterior cingulate cortex (frontal lobe); cACC, caudal anterior cingulate cortex (frontal lobe); PCC, posterior cingulate cortex (parietal lobe); ICC, isthmus cingulate cortex (parietal lobe); PHC, parahippocampal cortex; Th, thalamus; Amg, amygdala; Hipp, hippocampus; NAc, accumbens.

Highlight the existence of statistically significant differences.

**Table 4.** Regression analysis of protein intake (g/kg/w) and volume (mm^3^) of the cortical and subcortical regions within the limbic system in the first week of life, for school-aged children with a history of prematurity. Regression adjusted for child age and BMI in the structural analysis of MRf

Variable (mm^3^)	Not adjusted				Adjusted			
	b	se	R^2^	*P*	b	se	R^2^	*P*
rACC – L	16·5	13·0	0·022	0·208	22·6	18·9	0·040	0·235
rACC – R	–5·79	12·4	0·003	0·643	–12·7	18·2	0·011	0·488
cACC – L	8·057	10·5	0·008	0·446	3·96	15·2	0·028	0·796
cACC – R	0·692	14·7	0·000	0·963	–9·55	21·5	0·006	0·658
PCC – L	20·3	11·2	0·044	0·074	0·89	16·1	0·083	0·956
PCC – R	24·1	12·1	0·053	0·050	7·00	17·4	0·090	0·689
ICC – L	11·0	11·9	0·012	0·359	–13·3	17·0	0·066	0·437
ICC – R	22·1	9·5	0·069	**0·023**	8·46	13·8	0·094	0·544
PHC – L	–1·08	7·4	0·000	0·885	–1·82	10·9	0·012	0·868
PHC – R	4·09	7·3	0·004	0·577	5·10	10·6	0·014	0·634
Th – L	9·58	18·7	0·004	0·610	19·7	27·2	0·016	0·471
Th – R	9·39	18·1	0·004	0·606	13·5	26·4	0·019	0·610
Amg – L	4·83	4·8	0·014	0·324	8·47	7·1	0·021	0·237
Amg – R	6·36	5·2	0·020	0·229	8·32	7·6	0·023	0·282
Hipp – L	–0·90	8·9	0·000	0·920	5·47	13·1	0·009	0·678
Hipp – R	8·09	9·7	0·009	0·411	11·3	14·3	0·014	0·431
NAc – L	3·20	2·1	0·029	0·148	2·07	3·2	0·033	0·521
NAc – R	1·23	2·1	0·005	0·559	0·60	3·07	0·015	0·845

L, left hemisphere; R, right hemisphere. rACC, rostral anterior cingulate cortex (frontal lobe); cACC, caudal anterior cingulate cortex (frontal lobe); PCC, posterior cingulate cortex (parietal lobe); ICC, isthmus cingulate cortex (parietal lobe); PHC, parahippocampal cortex; Th, thalamus; Amg, amygdala; Hipp, hippocampus; NAc, accumbens.

Highlight the existence of statistically significant differences.

In the regression analysis, after adjusting for gestational age and BMI, we observed that lipid intake during the first week of life was associated with the volumes of the left thalamus (*b* = 50·7; *P* = 0·014), the right thalamus (*b* = 47·4; *P* = 0·018), the left nucleus accumbens (*b* = 5·95; *P* = 0·008) and the right nucleus accumbens (*b* = 5·02; *P* = 0·031) (Table 5).

**Table 5.** Regression analysis of lipid intake (g/kg/w) and volume (mm^3^) of the cortical and subcortical regions within the limbic system in the first week of life, for school-aged children with a history of prematurity. Regression adjusted for child age and body mass index in the structural analysis of MRf

Variable (mm^3^)	Not adjusted				Adjusted			
	b	se	R^2^	*P*	b	se	R^2^	*P*
rACC – L	17·2	13·5	0·022	0·206	16·4	14·5	0·038	0·262
rACC – R	–2·15	12·9	0·000	0·868	–2·60	14·0	0·005	0·853
cACC – L	8·21	10·9	0·008	0·454	5·83	11·7	0·030	0·620
cACC – R	16·3	15·1	0·016	0·284	15·6	16·4	0·016	0·344
PCC – L	25·5	11·5	0·064	**0·030**	17·5	12·2	0·109	0·155
PCC – R	24·8	12·5	0·051	0·052	16·2	13·2	0·107	0·225
ICC – L	5·95	12·4	0·003	0·634	–3·36	13·1	0·058	0·798
ICC – R	1·58	10·3	0·000	0·878	–8·81	10·6	0·098	0·410
PHC – L	1·79	7·77	0·001	0·818	2·20	8·3	0·012	0·794
PHC – R	11·9	7·4	0·034	0·114	12·7	8·0	0·044	0·119
Th – L	44·3	18·7	0·072	**0·020**	50·7	20·1	0·091	**0·014**
Th – R	42·9	18·1	0·072	**0·021**	47·4	19·5	0·092	**0·018**
Amg – L	3·25	5·0	0·006	0·523	3·25	5·4	0·006	0·555
Amg – R	7·91	5·4	0·029	0·149	7·66	5·8	0·030	0·196
Hipp – L	13·0	9·2	0·027	0·159	17·0	9·8	0·047	0·089
Hipp – R	18·9	9·9	0·048	0·062	20·1	10·7	0·052	0·067
NAc – L	5·95	2·1	0·093	**0·008**	5·52	2·3	0·096	**0·023**
NAc – R	4·87	2·1	0·069	**0·024**	5·02	2·2	0·079	**0·031**

L, Left hemisphere. R, right hemisphere; rACC, rostral anterior cingulate cortex (frontal lobe); cACC, caudal anterior cingulate cortex (frontal lobe); PCC, posterior cingulate cortex (parietal lobe); ICC, isthmus cingulate cortex (parietal lobe); PHC, parahippocampal cortex; Th, thalamus; Amg, amygdala; Hipp, hippocampus; NAc, accumbens.

Highlight the existence of statistically significant differences.

**Figure 3.**
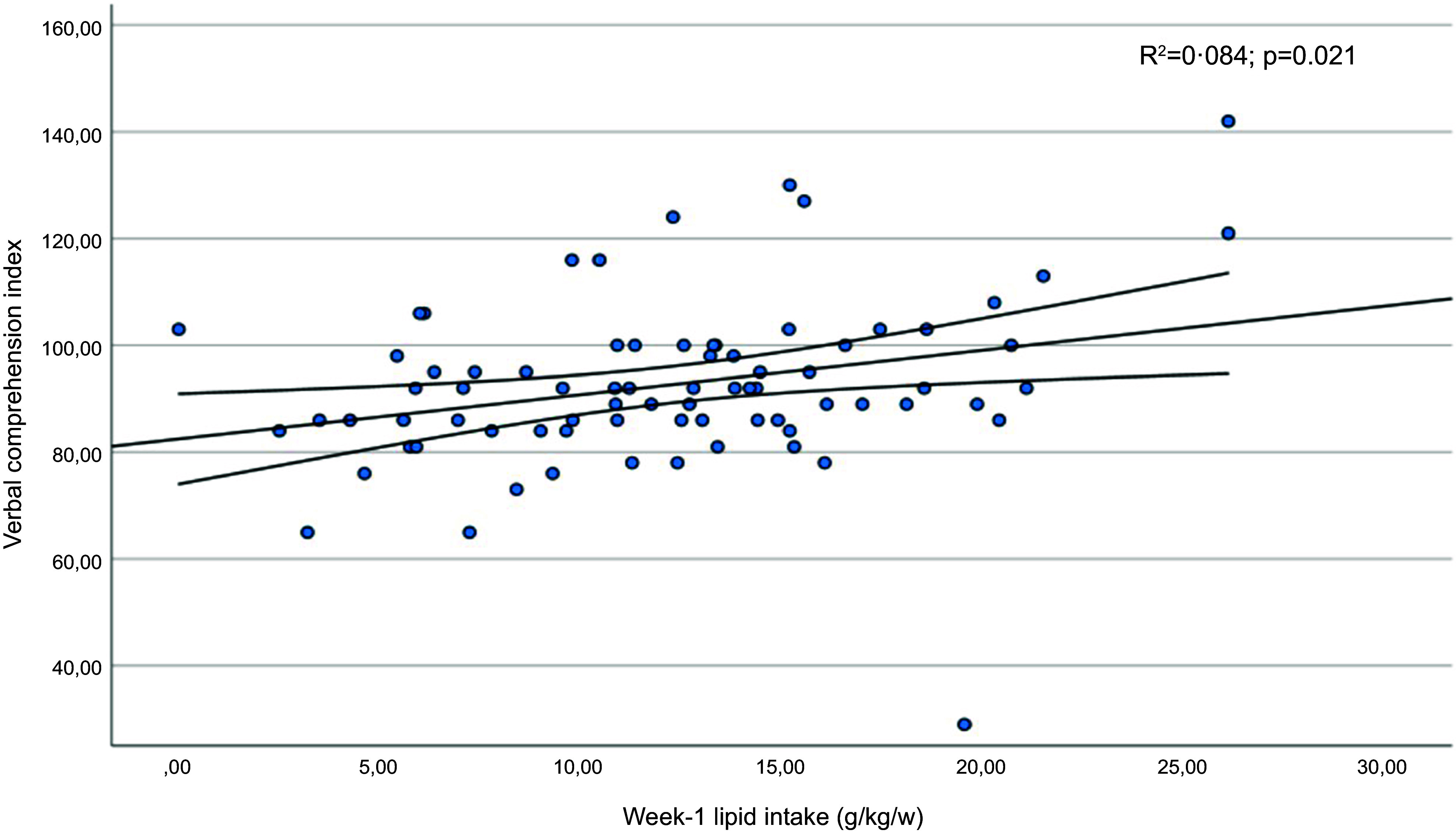
Regression diagram for verbal comprehension subdomain scores (WISC-V) and lipid intake in the first week of life. Regression adjusted for child age and body mass index in the structural analysis of MRf.

**Figure 4.**
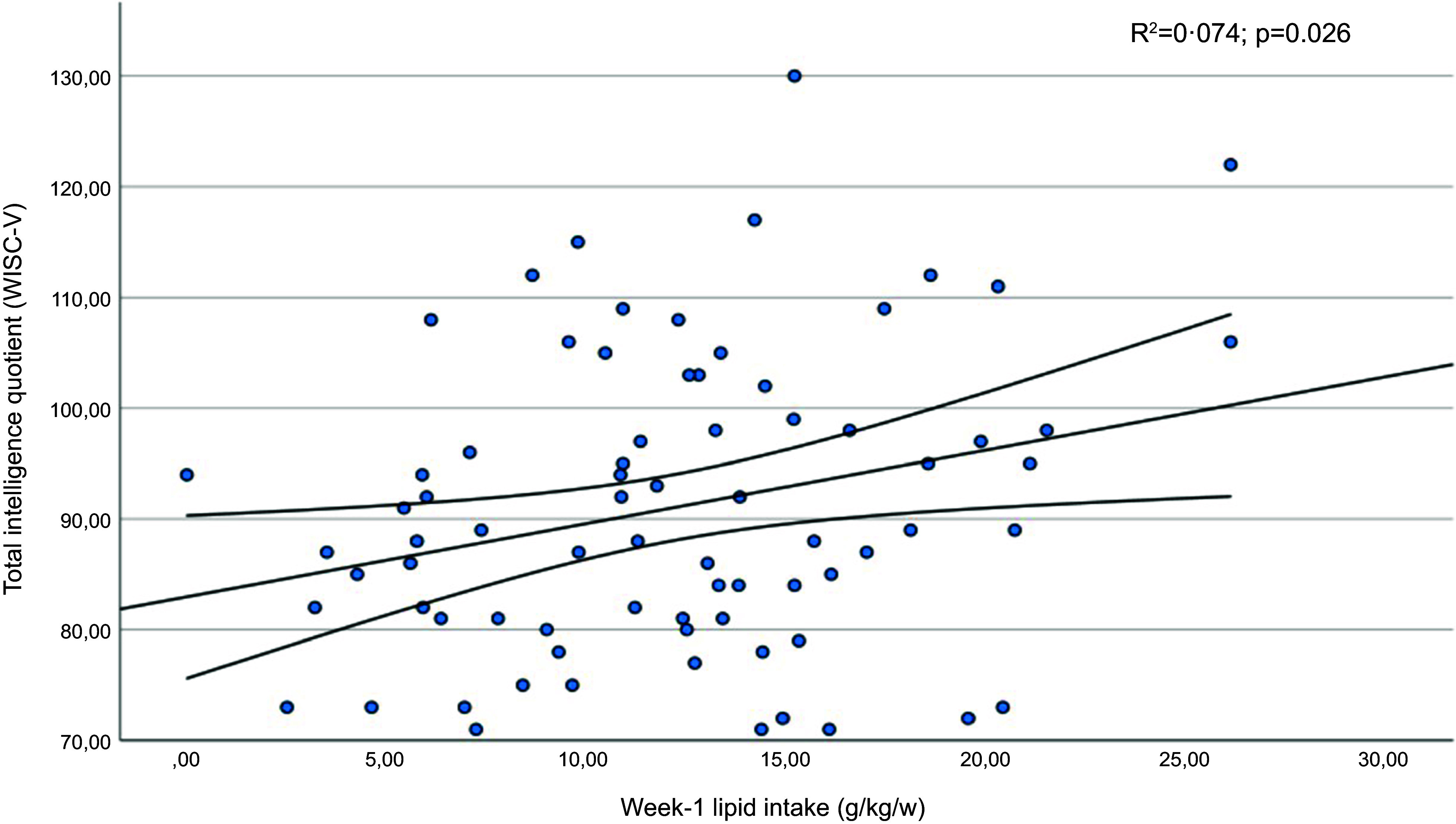
Regression diagram for intelligence quotient (WISC-V) and lipid intake in the first week of life. Regression adjusted for child age and BMI in the structural analysis of MRf.

Table 6 shows the mean scores obtained in the subdomains of the WISC-V test by children whose protein or fat intake was below the first quartile in the early neonatal period. Furthermore, significantly lower coefficients were recorded for the verbal comprehension index, the visuospatial index and the intelligence quotient in children whose lipid intake had been below the first quartile. The verbal comprehension index and intelligence quotient results presented a linear relationship with the infants’ lipid intake in the first week of life (Figures 3 and 4). In addition, statistically significant correlation coefficients were recorded between the volumes of the left and right thalamus, on the one hand, and the verbal comprehension index (*r* 0·305; *P* = 0·011 and *r* 0·322; *P* = 0·007, respectively) and the visuospatial index (*r* 0·298; *P* = 0·014 and *r* 0·252; *P* = 0·038, respectively).

**Table 6.** Scores obtained for the subdomains of the WISC-V with respect to energy, lipid and protein intake (total amount received in the first week of life), below and above the 1st quartile

	Lipids < Q_1_		Lipids > Q_1_		Protein < Q_1_		Protein > Q_1_	
	*n* 21		*n* 53		*n* 20		*n* 54	
	Mean	sd	Mean	sd	Mean	sd	Mean	sd
Verbal comprehension	87·3	11·6	95·4	17·0**	92·7	9·3	92·9	17·9
Visual-spatial	87·6	14·6	96·5	19·5*	93·0	17·9	93·9	18·9
Fluid reasoning	89·4	11·8	92·2	18·1	91·5	13·0	91·2	17·6
Working memory	83·9	10·9	86·5	16·8	83·5	9·5	86·5	16·9
Processing speed	101·2	11·8	97·2	17·8	101·9	12·1	97·1	17·5
Total IQ	86·2	10·9	93·1	14·0*	89·4	11·5	91·6	14·2

Mean (sd).

*P <0.05; **P <0.01.

There were no statistically significant associations between macronutrient intake in the early neonatal period of VLBW premature infants and the volumes of the amygdala, the parahippocampal cortex, the hippocampus or the anterior and rostral regions of the cingulate hemisphere.

## Discussion

In our study cohort of children born VLBW, the lipids intake in the first week of life was associated with the volume of various limbic system structures, including the thalamus and the nucleus accumbens. Another significant finding is that verbal comprehension and the ability to perceive, analyse and manipulate visual and spatial information may be impaired at the end of the first decade of life when the average lipid intake during the early neonatal period is below 1·2 g/kg/d. These findings are consistent with a growing body of evidence indicating that deficient macronutrient intake in VLBW preterm infants has long-term effects on brain development and functional outcomes during school age^(6,33)^.

Despite evidence of a strong association between premature birth, social vulnerability and adult psychopathology, the underlying biological mechanisms remain unclear, a knowledge gap that justifies further research to examine the relation between nutritional variables from the neonatal period and neurodevelopmental outcomes. In children with a history of extreme prematurity, neurodevelopmental and psychological issues, such as behavioural, cognitive and emotional disorders, can appear at school age and even later^(11)^. For example, the prevalence of autism spectrum disorder is much more frequent in these children than in those born full-term (8 % *v*. 0·6 %)^(34)^.

### Posterior cingulate cortex and the cingulate isthmus

The posterior cingulate cortex performs functions such as spatial orientation, visuospatial processing and narrative comprehension^(35)^. According to our analysis, the volume of this structure is associated initially with that of energy and lipid intake during the early neonatal period, although this association becomes non-significant when adjusting for chronological age and BMI. Aspects such as verbal comprehension and visuospatial index scored worse on the WISC-V test applied to school-age children when their lipid intake in the early neonatal period was lower. This association is to be expected, given that lipids intervene in neuronal development and in the differentiation and migration of nerve cells. This is of great importance during the first weeks of life for the correct functioning of the nervous system and for synaptogenesis^(36)^. The posterior cingulate cortex is connected by fibres to the anterior, ventral and intralaminar thalamic nuclei, the caudal portion of the temporoparietal junction and the superior temporal cortex^(37,38)^. The ventral section is involved in memory and thought processes, via the fibres that connect it with the medial temporal lobe, the prefrontal cortex and the hippocampus^(39)^. The dorsal portion participates in cognitive control and is activated during task performance; this region has fewer connections with the limbic and paralimbic regions^(4,35)^. It has been suggested that the anomalous development of the U-fibres of the posterior cingulate cortex may generate functional anomalies in this region and also in the default mode neuronal network, which is important for socio-emotional relationships and the symptomatology of autism spectrum disorders^(40)^,

The cingulate is connected to the parahippocampal cortex via the isthmus. A reduced volume of this isthmus is associated with a higher prevalence of attention deficit hyperactivity disorder^(41)^. Other researchers have suggested that the altered glutamatergic signalling arising from a reduction in the volume of the posterior cingulate gyrus may be associated with attention deficit hyperactivity disorder^(42)^.

### The thalamus and the basal ganglia

The thalamus and the basal ganglia are grey matter structures that form functional networks with the cortex through a cortical-basal ganglia-thalamus-cortical circuit, composed of both direct and indirect pathways^(43)^. The main nuclei are the striatum (caudate nucleus, putamen and nucleus accumbens) and the pallidum. Information from the cortex is received in the striatum and projected to the pallidum. In the direct pathway, inhibitory signals from the pallidum received via the internal segment release the thalamus from tonic inhibition, increasing excitatory feedback to the cortex through thalamo-cortical projections. In the indirect pathway, inhibitory signals from the external segment of the pallidum suppress the activation of the projections^(43)^. These brain structures modulate the activity of a wide range of motor, cognitive, affective and somatosensory functions. Between 27 and 37 weeks of gestational age, the volume of the basal ganglia and the thalamus expands threefold^(44)^. Moreover, according to previous research, in premature survivors the basal ganglia and the thalamus are smaller than in their full-term counterparts, both during the neonatal period^(44)^, and also in childhood^(45)^ and adolescence^(46)^. Currently, there are no studies that link total lipid intake during the early neonatal period in VLBW preterm infants to the volume of the thalamus and basal ganglia. A clinical trial conducted with eighty-six term newborns demonstrated that essential fatty acids, such as DHA and arachidonic acid, are associated with improved central nervous system development in mammals. The authors concluded that pregnant women who received DHA and arachidonic acid supplementation had infants with significantly larger volumes of total brain, grey matter, corpus callosum and cortex, compared with the control group^(47)^.

Some authors have observed that faster growth rates in the basal ganglia are associated with higher intelligence quotient scores, particularly for reading and academic performance^(48)^. These results are in line with our own observations of positive correlations between the left and right thalamic volumes and indices of verbal and visuospatial comprehension and with the finding that the structural and functional characteristics referred to may be associated with lipid intake during the early neonatal period.

The main limitation of the present study arises from the number of children who declined to participate in the study – almost a third of the cohort. Furthermore, of those who did participate, nearly 50 % did not wish to undergo a functional magnetic resonance imaging examination after their neuropsychological evaluation. On the other hand, the age at which the MRf studies were performed in our cohort ranged from 6 to 16 years, which required us to age-adjust our association analyses.

### Conclusions

In view of the study findings, we conclude that lipids intake in the first week of life of VLBW premature newborns is associated with larger volumes in specific areas of the limbic system, such as the thalamus and the nucleus accumbens. These changes are related to higher scores in verbal comprehension, visuospatial index and intelligence quotient in later childhood.
